# Environmental DNA Metabarcoding as a Means of Estimating Species Diversity in an Urban Aquatic Ecosystem

**DOI:** 10.3390/ani10112064

**Published:** 2020-11-07

**Authors:** Heather J. Webster, Arsalan Emami-Khoyi, Jacobus C. van Dyk, Peter R. Teske, Bettine Jansen van Vuuren

**Affiliations:** 1 Centre for Ecological Genomics and Wildlife Conservation, Department of Zoology, University of Johannesburg, Auckland Park, Gauteng 2006, South Africa; heather.joy.webster@gmail.com (H.J.W.); arsalane@uj.ac.za (A.E.-K.); prteske@uj.ac.za (P.R.T.); 2 Department of Zoology, University of Johannesburg, Auckland Park, Gauteng 2006, South Africa; cvandyk@uj.ac.za

**Keywords:** environmental DNA, metagenomics, species diversity, biodiversity monitoring

## Abstract

**Simple Summary:**

Cities are the fastest developing ecosystems on the planet. The rapid expansion of urban areas is typically seen as a threat to global biodiversity, yet the role of cities in protecting species that may be rare in the wild remains poorly explored. Here, we report the use of environmental DNA (eDNA) to document the species present in one of the largest urban green spaces in Johannesburg, South Africa. We document a surprisingly large number of taxonomic groups, including some rare and threatened species. Our results support the notion that urban green spaces can provide refuge to a large number of species, and the species inventory provides critical information that can be used by city parks managers to conserve green spaces.

**Abstract:**

Adaptation to environments that are changing as a result of human activities is critical to species’ survival. A large number of species are adapting to, and even thriving in, urban green spaces, but this diversity remains largely undocumented. In the current study, we explored the potential of environmental DNA (eDNA) to document species diversity in one of the largest green spaces in Johannesburg, South Africa. Using a novel metabarcoding approach that assembles short DNA fragments suitable for massively parallel sequencing platforms to the approximate standard ~710 bp COI barcoding fragment, we document the presence of 26 phyla, 52 classes, 134 orders, 289 families, 380 genera and 522 known species from the study site. Our results highlight the critical role that urban areas play in protecting the world’s declining biodiversity.

## 1. Introduction

Urban areas are the fastest growing ecosystems on the planet. As cities expand, the surrounding natural habitat is transformed, and wild species are either displaced or are forced to persist in shrinking pockets of fragmented habitats inside cities [1,2,3]. As a consequence, parks, green spaces, architectural structures and wetlands within cities harbor a considerable level of concealed diversity [4,5,6,7] whose significance remains largely underappreciated. In cities, species face a combination of environmental stressors and selective anthropogenic pressures that differ from those found in natural environments [8]. However, for those species that can adapt to the challenges of living in urban areas, there are numerous benefits. Constant supply of food, shelter, fewer predators or competitors, and more stable micro-climatic conditions make cities an ecological hotspot for many species [9]. Species that have mastered living in urban areas can reach population densities that far exceed those of their conspecifics in the wild, making urban ecosystems particularly important for the survival of numerous threatened species across the globe [10,11,12].

Johannesburg is the largest and most populated metropolitan area in South Africa [13]. Founded in the mid-1880s during the Witwatersrand gold rush, the city is situated in the Gauteng Province within the eastern Highveld plateau ecoregion [14] of South Africa. Our study area, Delta Park, is one of the largest semi-natural green spaces in Johannesburg. Until the 1960s, Delta Park was used as an expansion of Johannesburg’s sewage scheme. Soon after the closure of the sewage site, an ecosystem restoration project started, and over time, several species colonized the area, with at least 200 bird species documented in Delta Park by 2011 [15,16]. However, the diversity of less conspicuous taxa that are associated with water, such as arthropods and mollusks, remains unexplored.

Traditional methods of monitoring biodiversity rely on visual observation and morphological identification of species that live in an area [17,18]. These approaches require extensive taxonomical and morphological expertise, and are unsuitable to monitor species that are rare, cryptic, secretive [19], or that change phenotypically throughout their lives [18,20].

Recent developments in DNA sequencing technologies and high-performance computing have made it possible to study ecological diversity with unprecedented levels of accuracy. Particularly suitable for monitoring the diversity and abundance of species is the massively parallel sequencing of environmental DNA (eDNA) [18], which is a non-destructive approach [21] that utilizes trace amounts of DNA found in the environment [22]. This technique is highly sensitive and has considerable potential to identify scarce, cryptic, or elusive species that are otherwise overlooked [23].

Most DNA-based methodologies are sensitive to the selection of appropriate DNA markers. Elbrecht and Leese (2015) [24] showed that the selection of genetic markers directly influences the estimation of species diversity and abundance in an area. Since 2002, the cytochrome c oxidase subunit I gene (COI) has been the marker of choice for DNA-based biomonitoring [25]. The performance of this marker has been tested in a wide range of freshwater, marine, and terrestrial habitats [26,27,28,29,30,31,32,33] to address a wide array of ecological questions [34,35,36,37].

The approximate 710 bp length of the COI marker of Folmer et al. (1994) [38], which is the most frequently amplified fragment, exceeds the current limits of most massively parallel sequencing platforms. While new sequencing platforms that produce longer sequences, such as PacBio and Oxford Nanopore technologies exist, the high costs of sequencing on these platforms makes the technology inaccessible to many laboratories in lower income countries. As an alternative, new sets of DNA primers that target shorter segments of DNA than the universal primers of Folmer and colleagues have been developed [39,40,41,42]. A trade off is that a combination of several barcodes is typically required to study the total diversity of an area (i.e., taxon/species-specific primers for different taxonomic groups), which involves extra effort and additional costs for optimizing amplification and sequencing.

The current study explores the potential of randomly shearing COI sequences into short fragments, followed by bioinformatic assembly of the complete length of the targeted product of Folmer’s primers. The aims of this study are two-fold. First, it constitutes the first DNA-based survey of species diversity in a South African urban green space, five decades after an ecological restoration project started. Second, we present a cost-effective method for monitoring biodiversity, which uses short shotgun fragments from commonly used sequencing platforms combined with high-throughput bioinformatic pipelines, to approximately the complete ~710 bp length of the COI barcode. The same methodology can be applied to reconstruct the full length of any other DNA fragments.

## 2. Materials and Methods

### 2.1. Study Area

**Figure 1:**
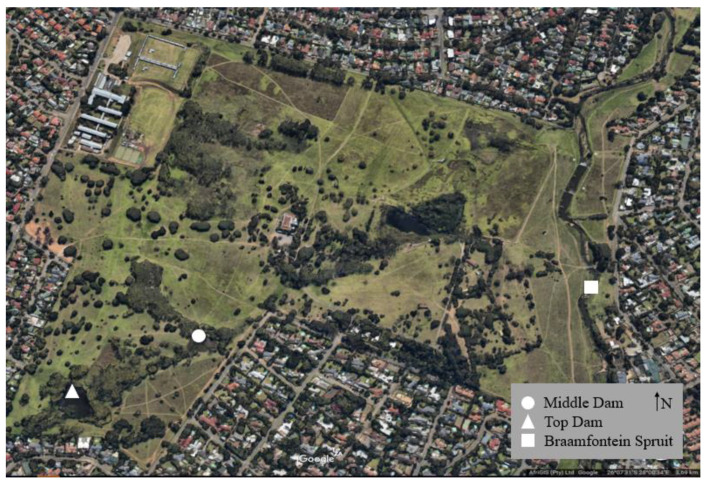
Sampling localities within Delta Park, Johannesburg. The Braamfontein Spruit flows in a northerly direction. The three collection sites are indicated. Aerial photo taken from Google Earth.

Delta Park is located along the middle reaches of the Braamfontein Spruit (river), a tributary of the Jukskei River which falls within the larger Crocodile (West) and Marico Water Management Area. The upper reach of the river flows through urban areas in the west of Johannesburg while the middle reach of the Braamfontein Spruit is surrounded by formal residential areas. The two dams in the park (Top Dam and Middle Dam) are connected by a narrow channel whose water flows in a north-easterly direction through the park before it joins the Braamfontein Spruit (see Figure 1).

### 2.2. Sample Collection

Water and sediment samples were collected from three sites within Delta Park: Middle Dam (−26.127914, 28.006980), Top Dam (−26.129288, 28.003892) and the Braamfontein Spruit (−26.126860, 28.016090) (Figure 1). At each site, water was collected in two 500 mL bottles that had been sterilized through autoclaving (KT—2346A, ALP Co., Ltd. Tokyo, Japan) for 40 min at 115 °C under 1 atmosphere pressure. At the collection site, the lids were opened approximately 15 cm below the water surface, the bottles were filled with water, and the lids were closed while still underwater. For sediment collection, soil was collected into three 50 mL plastic tubes from various sites in the dams and river, approximately 1 m from the bank. All samples were immediately transferred to the Centre for Ecological Genomics and Wildlife Conservation at the University of Johannesburg. Water samples were placed in a refrigerator at 4 °C and sediment samples were preserved in a freezer at −20 °C. Environmental samples were processed within 24 h of collection.

Environmental samples were processed in a room that has not been used for any DNA work prior to this study. All surfaces were wiped down with 100% bleach, left to dry, and subsequently wiped with 70% EtOH. Approximately 500 mL of the collected water was filtered using a MicroFunnelTM Filter Unit with 0.2 µm Supor^®^ Membrane (Pall Laboratory, Johannesburg, South Africa). Five filters were used for the Middle Dam site, two filters were used for Top Dam, and three filters were used for the Braamfontein Spruit. We changed filters when they became clogged, and DNA was extracted from all the filters. For the sediment, each falcon tube was shaken vigorously and approximately 5 mg of the homogenized slurry was subsampled for DNA extraction.

Metagenomic DNA was extracted by grinding sediment and water filters in 200 µL grinding buffer (0.5 M sorbitol, 0.2 M Tris-HCL, 7 mM TITRIPLEX^®^ III EDTA, 20 mM Na-Bisulfit and 4% polyvinylpyrrolidone 40) using a Covaris sonicator (Whitehead Scientific, Cape Town, South Africa). Ground specimens were digested using 200 µL of lysis buffer (0.4 M Tris-HCL, 7 mM TITRIPLEX^®^ III EDTA, 2 M NaCl and 2% of cetrimonium bromide), 10% SDS and 20 µL Proteinase K in a 1.5 µL Eppendorf tube. The samples were left to incubate for one hour at 60 °C. Lysates were centrifuged (4600 RPM) for 10 min at 4 °C. The supernatant was transferred into a new 1.5 µL Eppendorf tube, and 250 µL buffer mix III (3 M KAc) was added to each tube. The tubes were left to incubate on ice for 10 min followed by a 15 min centrifugation step at 4 °C. The supernatant was transferred onto a glass fibre filter plate and was centrifuged for an additional 5 min at 4 °C. Approximately 280 µL of isopropanol was added to the filtrate, which was then vortexed vigorously and left to incubate on the plate for 20 min at −20 °C. Each plate was centrifuged for an additional 25 min at 4 °C. Finally, 200 µL of ice cold 70% EtOH was added to each tube, which was centrifuged for 5 min at 4 °C. The resulting DNA pellets were dried at room temperature and then dissolved in 50 µL TE buffer.

A portion of the mitochondrial COI gene was amplified using 3 µL of template DNA, 6 µM of universal primers LCO1490 (forward primer, 5′–GGT CAA CAA ATC ATA AAG ATA TTG G–3′) and HCO2198 (reverse primer, 5′–TAA ACT TCA GGG TGA CCA AAA AAT CA–3′) [38], 1× PCR buffer, 0.4 mM dNTPs, 2.5 mM MgCl_2_, and 1 unit of Taq DNA Polymerase (S7 Phusion; Biozym, Oldendorf, Germany). Thermal cycles for each reaction started with an initial denaturation step at 95 °C for 15 min, followed by 25 cycles of denaturing steps at 95 °C for 20 s, an annealing stage of 49 °C for 45 s, and an elongation step at 72 °C for 1 min. This was followed by a final elongation step at 72 °C for 10 min. To minimize the amplification of non-specific PCR product, we selected 25 PCR cycles, as preliminary analyses indicated that this produced sufficient product for Illumina library preparations.

Equimolar concentrations of amplicons from sediment and water samples were pooled into two separate tubes and randomly sheared into approximately 250–300 bp fragments using a Covaris sonicator (Whitehead Scientific, Cape Town, South Africa). To maximize the random shearing of the COI amplicons, multiple rounds of sonication with different intensities were performed, and the size of the resulting fragments after each round of sonication was checked using a Bioanalyzer 2100 (Agilent, Johannesburg, South Africa). Only fragments within the expected range were selected for genomic DNA library preparation. The targeted sequence was treated as a miniature genome that was sheared to a large number of smaller overlapping fragments that is necessary to assemble longer fragments.

Genomic libraries were generated using a NEBNext^®^ UltraTM DNA Library Prep Kit (New England BioLabs, Ipswich, MA., USA) and sequenced on an Illumina HiSeq 4000 platform (San Diego, CA, USA) using 2 × 150 bp paired-end chemistry according to the manufacturer’s instructions.

### 2.3. Sequence Assembly and Analysis

Low-quality sequences and adapter contaminants were identified in FastQC [43] and removed using Trimmomatic v0.39 [44]. MEGAHIT v1.1.1 [45] was used to assemble metagenomic sequences into longer contigs using the program’s default settings. This metagenome assembler was chosen because it performs robustly in large and complex datasets that are typical of environmental samples [44]. Assembly statistics were estimated in QUAST v4.0 [46]. To check the quality of assemblies, the short read aligner Bowtie2 [47] was used to map quality-filtered sequences against corresponding assembled contigs, and the mapping statistics for each alignment were computed in SAMtools v1.10 [48].

The assembled sequences were dereplicated into unique sequence features using VSEARCH v2.4.2 [49], chimeric reads were removed, and the remaining reads were subsumed into distinct clusters known as operational taxonomic units (or OTUs), by executing a VSEARCH smallmem command with a minimum sequence similarity of 98%. Consensus sequences from each cluster were extracted and sorted based on their size. Taking into account variation in the primers’ annealing sites between different taxa, and minor length variation that is typically observed in metagenome assemblies from complex communities, all sequences with a length exceeding 712 bp (the theoretical maximum product size length for the LCO1490 and HCO2198 primers is approximately 710 bp [38]) were considered assembly artefacts or non-target sequences and filtered from downstream analyses.

All consensus sequences were searched against an in-house database of COI sequences extracted from the NCBI non-redundant nucleotide database (https://www.ncbi.nlm.nih.gov/refseq/), using the MEGABLAST package (which searches for ‘highly similar’ matches) [50] and somewhat similar BLASTn (which searches for ‘somewhat similar’ matches), with a minimum sequence similarity of 70% and an e-value of 10^−5^. The four best matches for each query were retained and reported. A Last Common Ancestor (LCA) consensus taxonomic rank was assigned to each sequence in BASTA v1.3.2.3 (https://github.com/timkahlke/BASTA) [51]. For each match, the NCBI taxonomy ID and scientific name were extracted, and a circular phylogenetic tree was constructed using the PhyloT online server (phylot.biobyte.de) [52] and visualized in FigTree v1.4.4 [53].

To assess the efficiency of the sequence assembly and taxonomic rank assignments, all sequences in our dataset were divided into two approximately equally sized fragments using FASTX-Toolkit v 0.0.8 [54]. Small overlaps were allowed between two fragments, especially for shorter fragments (<400 bp). Each fragment was separately blast-searched using the same parameter settings for e-value and percentage identity as for the full-length dataset. The BASTA pipeline relies on unique NCBI accession numbers to assign a consensus taxonomic rank to the query sequences. To account for variations in NCBI accession numbers, an issue which typically arises when the first half of the sequence matches a specific accession number of a species and the second half matches the same species but from an entry with a different accession number, we verified whether the NCBI accession numbers for the best matches of the first half can be exactly matched among the four best matches reported for the second half. Furthermore, a database consisting of 100 chimeric sequences was manually created by adding random fragments of DNA from multiple arthropods and vertebrate species, and all these sequences were subjected to the same taxonomic rank assignments.

Alpha diversity indices, Shannon [55], Simpson [55] and Evenness [56], were estimated using the R package diverse [57].

## 3. Results

**Figure 2:**
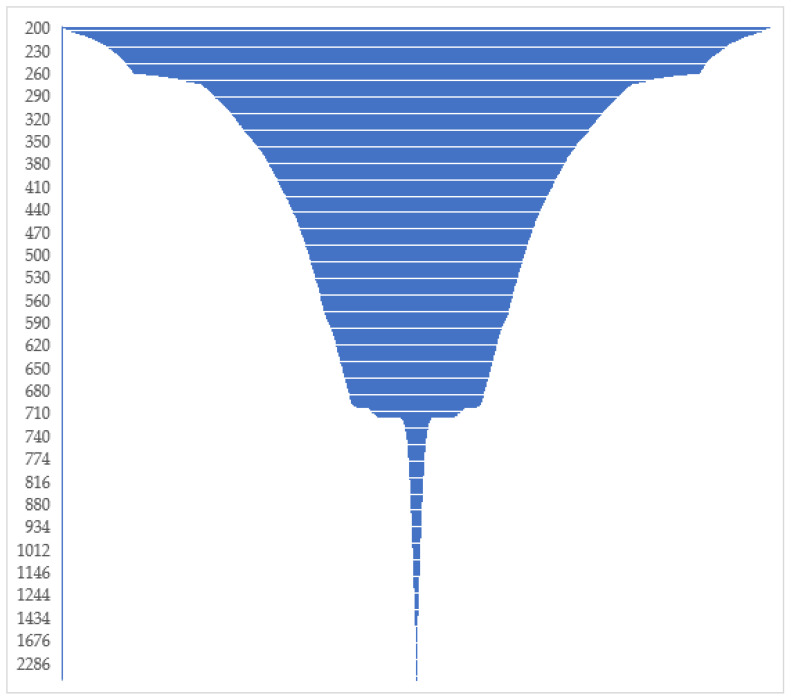
A funnel graph showing the cumulative length distribution of the unfiltered assembled sequences. The Y axis represents the cumulative length of the scaffolds in base pair. The bottleneck in the figure reflects ~710 bp, the theoretical length of the target sequences. Less than 5% of sequences had a length exceeding 713 bp, and these were removed from the dataset.

The Illumina sequencing run yielded 5,028,734 and 5,219,475 paired-end raw sequences from water and sediment samples, respectively. MEGAHIT assembled quality-filtered sequences from water samples into 2208 and 5443 contigs, respectively. The cumulative length distribution histogram of raw assemblies shows that the length of less than 5 percent of the sequences exceeded 712 bp (Figure 2).

**Figure 3:**
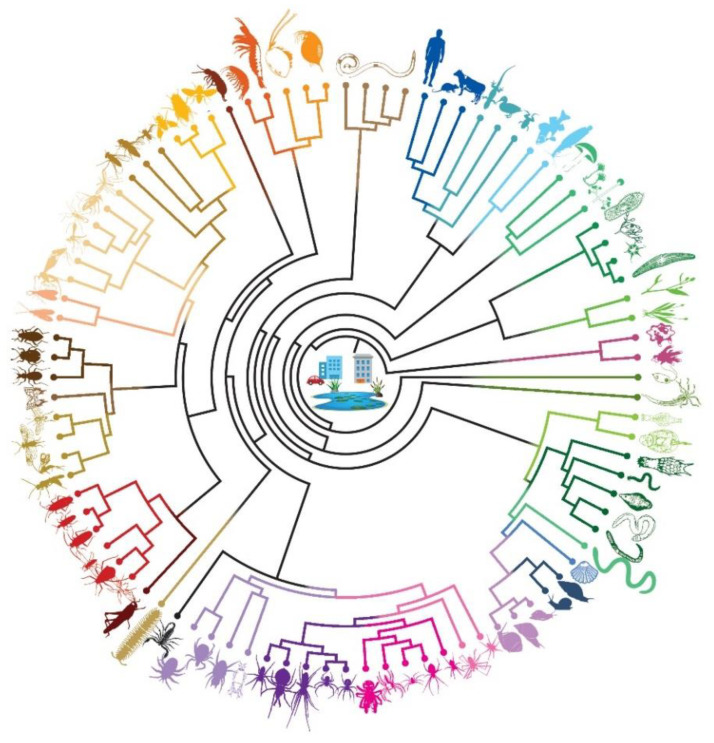
A circular tree showing the different organisms found in the water and sediment samples in Delta Park, South Africa. This tree is based on the results from the BLASTN search results.

After merging raw assemblies from water and sediment, VSEARCH dereplicated the pool into 6319 unique features. Subsequent clustering of unique sequence features with more than 98% identity produced 5582 clusters. The BASTA pipeline taxonomically ranked the resulting dataset, with a mean blast percentage identity of 85% (range 72–100), into 26 phyla, 52 classes, 134 orders, 289 families, 380 genera, and 522 known species. Among these, only 12 species, namely *Achlya bisexualis, Aspergillus tubingensis, Biomphalaria glabrata, Bulinus natalensis*, *Cheyletus malaccensis, Chrysomya rufifacies, Drosophila hydei, Fannia canicularis, Homo sapiens, Opistophthalmus boehmi, Rattus norvegicus,* and *Tuberolachnus salignus*, are known to occur in Delta Park based on earlier, non-genetic studies (Table 1, Figure 3; see Appendix A Table A1 for a complete taxonomic list). Our results show that the taxonomic rank assignment is sensitive to the selection of the blast algorithm, as 35.9% of species identified by highly sensitive MEGABLAST were absent when we used BLASTn. Similarly, 9.7% species that were identified by BLASTN were absent in MEGABLAST (Appendix A). More than 70% of the quality-filtered reads were properly mapped against the corresponding assemblies, which lies within the accepted range for an assembly.

The exact NCBI accession number of the best matches for the first half of the assembled sequences were matched among four best matches of the second half in more than 98.4% of the pair-wise comparisons. In almost all cases, the exact match for NCBI accession number of the first half was found among the top eight blast hits for the second half. The BASTA pipeline assigns a taxonomic rank of “unknown” or “unknown-eukaryotic” to all manually generated chimeric sequences. All these confirm that the negative effects of chimeric sequences in our dataset are likely to be minimal.

Shannon’s diversity index was estimated slightly higher for sediment compared to water samples (sediment H = 2.13 and water H = 2.025), while both the Evenness index and the Simpson’s index were higher for the water communities (Table 2).

## 4. Discussion

Cities constitute the newest extensions to wild habitats. The role that urban ecosystems play in preserving the world’s declining biodiversity represents an underappreciated area of research [58], yet it remains critical in ensuring the conservation of many species and the provisioning of ecosystem services. Living alongside wild populations exposes societies to health challenges; these challenges need to be addressed proactively using a combination of conventional and new tools. Here, we present and discuss the first application of environmental DNA to survey biological diversity in a semi-natural park in Johannesburg.

Not surprisingly given their abundance, arthropods dominated the aquatic biological diversity of Delta Park. Arthropods were followed by species belonging to the phyla Oomycota, Mollusca, Cnidaria, Chordata, Rotifera and Annelida. High diversity of arthropods has already been reported from urban areas in South Africa [59] and elsewhere [60]. Among arthropods, the presence of the assassin spiders, Archaeidae, is of particular interest. With only one extant genus, *Afrarchaea*, reported from South Africa [61], their presence in Delta Park highlights the importance of urban ecosystems for the survival of species that are comparatively rare in wild habitats [52,53,54,55,56,57,58,59,60,61,62,63,64,65].

Delta Park is home to several firefly species. Populations of fireflies are declining across the globe as a result of high intensity artificial light at night (ALAN) [66,67,68] that exceeds the intensity of bioluminescent flashes of these nocturnal species during the mating season. The lower level of artificial light contamination in Delta Park compared to the densely populated surrounding residential areas makes it an ideal breeding habitat for this ecologically important species. The presence of members of Culicid mosquitos and Tabanid horse flies, among which there are several species that function as biological vectors for the causative agents of some diseases such as malaria, yellow and dengue fever, are also important.

Environmental DNA highlighted the presence of several vertebrate species in Delta Park. These include rats (*Rattus* sp.), cattle (*Bos taurus;* not physically present in the park, but likely the result of DNA carried by water), and a number of unidentified species belonging to Gekkonidae, Scincidae and Eulipotyphla families (all present in the park). Among aquatic vertebrates, species of Perciformes, Blenniidae, Cypriniformes, *Clarias* sp. and Galaxiidae fish were identified. While some of these species such *Clarias* sp. are common inhabitants of freshwater ecosystems worldwide and in southern Africa, the presence of some other species most likely represents non-native species released into dams by aquarium owners.

Delta Park is home to two species of freshwater aquatic snails, *Bulinus natalensis* and *Biomphalaria glabrata*. Both are intermediate hosts for parasitic flatworms (*Schistosoma* sp.). Infection with schistosome flatworms can progress to the development of schistosomiasis, the world’s third most devastating parasitic disease [69]. The identification of a pathogenic protist, *Acanthamoeba* sp., the causative agent for ocular keratitis and granulomatous encephalitis of the central nervous system, is also a major health concern [70].

The primers used in the current study were initially developed for animals. However, the identification of the several species of fungi and algae in our dataset points to the potential of the COI marker for the identification of additional taxa, although only at higher taxonomic ranks [71], because in line with previous studies [72], there was little taxonomic resolution at lower taxonomic levels such as genus and species level. We demonstrated that environmental DNA obtained from both water and sediment samples can be used to estimate species diversity in a wetland even if species are present at low numbers and were not detected during visual surveys. Our study had higher biodiversity compared to another river in South Africa (H′ = 1.028) [73], and was similar in comparison to a study on an urban temporary pond (H′ = 2.72) [74], and that of fish in large rivers (H′ = 2.21) [75]. This is because the environment can retain a molecular imprint of species inhabiting the area [76,77]. Determining when the species were present in the system is difficult, and it cannot be reliably expressed whether the species identified are still present in the system. Our study is a snapshot of the eDNA that was present in the system at the time of sampling, and over time, temporal communities can change in response to seasons and water quality, which needs to be a focus of future studies.

While we cannot rule out that some of the sequences that have failed conclusive assignment to lower taxonomic ranks are artefacts of the bioinformatic assembly, others undoubtedly reflect the lack of publicly available records for the species in question, or for their close relatives.

Methods that are based on the last common ancestor to assign taxonomic rank perform poorly when local databases are not complete, which is a common problem in underrepresented geographic areas such as Africa [78], as well as when the geographical records of the specimens in a database are heterogeneous. In both cases, conservative rank assignments to a higher taxonomic rank or to a taxonomic rank that does not occur within the study area will negatively affect the accuracy of such surveys. It is expected that underrepresented taxa and regions will be affected disproportionately.

As scientific efforts to characterize global biodiversity using environmental DNA intensify, our study emphasizes that the need for a comprehensive taxonomically-curated reference database is equally important. A reference database to address such shortcomings requires close cooperation between experts from different fields, such as systematics, morphology, biochemistry and molecular biology, at both regional and global scales. Successful collaborations can have far-reaching implications for better characterizing global biodiversity [79].

## 5. Conclusions

We demonstrated that environmental DNA obtained from water and sediment samples can be used to detect the presence of species in aquatic habitats, even if a species is present at densities undetected during visual biodiversity surveys. This reaffirms that aquatic habitats retain a molecular imprint of species inhabiting the area [71]. However, determining when the species were present in the system cannot be reliably assessed.

The specific methodology applied in the current study, which is based on the sequence assembly, results in different sections of the markers being covered with different number of raw sequences. This heterogeneous coverage across the length of the amplicon limits the power of such methodologies to estimate the abundance of each species. However, presence or absence of species in an area can be reliably investigated.

## Figures and Tables

**Table 1:** Number of sequences identified at different taxonomic ranks for the water and sediment samples combined.

Taxonomic Group	Phylum	Class	Order	Family	Genus	Species	Total
Annelida	1	2	4	7	12	16	42
Arthropoda	1	8	31	102	126	116	384
Ascomycota	1	3	2	4	2	3	15
Bacillariophyta	1	2	4	5	3	6	21
Basidiomycota	1	1	2	2	1	1	8
Bryozoa	1	1	1	1	1	1	6
Chordata	1	5	14	21	22	21	84
Ciliophora	1	1	1	1	1	1	6
Cnidaria	1	3	10	25	25	24	88
Discosea	1	1	3	4	6	18	33
Echinodermata	1	4	6	9	10	8	38
Evosea	1	1	1	1	2	2	8
Gastrotricha	1	0	1	1	1	1	5
Heterolobosea	1	0	0	1	1	0	3
Mollusca	1	2	10	24	28	26	91
Mucoromycota	1	1	1	1	1	0	5
Nematoda	1	1	2	3	3	2	12
Nemertea	1	2	2	2	2	2	11
Ochrophyta	1	3	9	17	41	66	137
Onychophora	1	1	1	2	2	2	9
Oomycota	1	2	4	5	12	96	120
Porifera	1	2	5	8	5	5	26
Rhodophyta	1	2	15	32	55	71	176
Rotifera	1	2	2	8	12	29	54
Streptophyta	1	1	2	2	2	1	9
Tubulinea	1	1	1	1	4	4	12

**Table 2:** Diversity indices showing Simpson Diversity, Shannon Diversity and Evenness.

Environmental Sample	Simpson D	Simpson I	Simpson R	Shannon	Evenness
Water	0.188	0.812	5.318	2.025	0.730
Sediment	0.181	0.819	5.522	2.130	0.689

## Appendix A

**Table A1:** Comparison of species identified using two different blast algorithms: highly sensitive MEGABLAST and BLASTn.

Species	BLASTN	MEGABLAST
*Acanthamoeba*	Yes	Yes
Achatinellidae	Yes	Yes
*Achlya*	Yes	Yes
*Achlya bisexualis*	Yes	Yes
Acrididae	Yes	No
Actinopteri	Yes	Yes
Agaricomycetes	Yes	Yes
Agelenidae	Yes	Yes
*Agelenopsis*	Yes	Yes
*Aglaoctenus*	Yes	No
Agromyzidae	Yes	Yes
*Alona*	No	Yes
Amaurobiidae	Yes	Yes
*Amaurobioides*	Yes	Yes
Amphibia	Yes	Yes
Amphipoda	Yes	Yes
*Anacaena*	No	Yes
Anatidae	Yes	No
*Anelosimus*	Yes	Yes
Anisakidae	Yes	Yes
Annelida	Yes	Yes
Anthocoridae	Yes	No
Antrodiaetidae	Yes	Yes
Anura	Yes	Yes
*Aphelenchoides*	Yes	Yes
Aphelenchoididae	Yes	Yes
Aphididae	Yes	Yes
*Apodemus*	No	Yes
Arachnida	Yes	Yes
Araneae	Yes	Yes
Araneidae	Yes	Yes
Archaeidae	No	Yes
Arthropoda	Yes	Yes
Ascomycota	Yes	Yes
*Aspergillus tubingensis*	Yes	No
Atemnidae	No	Yes
*Atomaria*	No	Yes
*Aulodrilus*	No	Yes
Aves	Yes	No
Bacillariaceae	Yes	Yes
Bacillariophyta	Yes	Yes
Basidiomycota	Yes	Yes
Bdelloidea	Yes	Yes
*Bellamya*	No	Yes
*Biomphalaria glabrata*	Yes	Yes
Bivalvia	Yes	Yes
Blenniidae	No	Yes
Boletaceae	No	Yes
*Bos taurus*	Yes	Yes
*Bosmina*	Yes	Yes
*Bourletiella*	No	Yes
Bovidae	Yes	Yes
*Bracon*	Yes	Yes
Braconidae	Yes	Yes
*Branchiodrilus*	Yes	Yes
Branchiopoda	Yes	Yes
*Bulinus natalensis*	Yes	Yes
Buthidae	Yes	Yes
Calliphoridae	Yes	No
Camisiidae	No	Yes
Caponiidae	No	Yes
Carabidae	Yes	Yes
Caraboctonidae	Yes	Yes
Cecidomyiidae	Yes	Yes
Cerambycidae	No	Yes
Ceratopogonidae	Yes	Yes
Chaetocerotales	No	Yes
Chaetonotida	Yes	Yes
Chaoboridae	No	Yes
*Cheiracanthium*	Yes	Yes
*Cheyletus*	Yes	Yes
*Cheyletus malaccensis*	Yes	No
Chironomidae	Yes	Yes
*Chlaenius*	No	Yes
Chlorophyta	Yes	Yes
Chloropidae	Yes	Yes
Chordata	Yes	Yes
Chromadorea	Yes	Yes
Chrysomelidae	Yes	Yes
*Chrysomya rufifacies*	No	Yes
*Chrysoperla*	Yes	Yes
Chrysophyceae	Yes	Yes
Chydoridae	No	Yes
Cicadellidae	No	Yes
Cicadidae	No	Yes
Ciliophora	Yes	Yes
*Clarias*	Yes	Yes
Clitellata	Yes	Yes
Clubionidae	No	Yes
*Cochliopodium*	Yes	Yes
Coleoptera	Yes	Yes
Colletidae	No	Yes
Columella	No	Yes
*Conioscinella*	No	Yes
*Contracaecum*	No	Yes
Corinnidae	No	Yes
Corydalidae	Yes	No
*Cotesia*	Yes	Yes
*Cottus*	No	Yes
Crabronidae	Yes	Yes
Crambidae	Yes	Yes
*Cricotopus*	Yes	No
Cryptophyceae	Yes	Yes
*Culex*	No	Yes
Culicidae	Yes	Yes
Cybaeus	Yes	No
Cyclopidae	Yes	Yes
Cyclopoida	Yes	Yes
Cypriniformes	No	Yes
*Daphnia*	No	Yes
Daphniidae	Yes	Yes
Decapoda	Yes	Yes
*Dictyuchus*	No	Yes
*Dinotrema*	No	Yes
Diplopoda	Yes	Yes
Diptera	Yes	Yes
Discosea	Yes	Yes
Dolichopodidae	Yes	No
Dorylaimida	Yes	Yes
*Drosophila*	Yes	Yes
*Drosophila hydei*	Yes	Yes
Drosophilidae	Yes	Yes
Elateridae	No	Yes
Ellobiidae	No	Yes
*Ellobium*	No	Yes
*Empoasca*	No	Yes
Endomychidae	Yes	No
Enoplea	Yes	No
*Enoplognatha*	No	Yes
*Entomobrya*	No	Yes
Entomobryomorpha	Yes	Yes
*Erigone*	No	Yes
Eulipotyphla	No	Yes
Eunotiales	No	Yes
Euonychophora	No	Yes
*Fannia canicularis*	Yes	No
*Folsomia*	Yes	Yes
Formicidae	No	Yes
Galaxiidae	No	Yes
Gastropoda	Yes	Yes
Gastrotricha	Yes	Yes
*Geckolepis*	No	Yes
Gekkonidae	Yes	Yes
Geometridae	No	Yes
Geomitridae	Yes	Yes
Glomerida	No	Yes
*Glyptapanteles*	No	Yes
Gnaphosidae	Yes	Yes
*Gyponana*	No	Yes
Haplotaxida	Yes	Yes
*Helobdella*	Yes	Yes
Hemiptera	Yes	Yes
Heptageniidae	No	Yes
Heterolobosea	Yes	Yes
Heteronemertea	Yes	Yes
Himatismenida	Yes	Yes
Hirudinida	Yes	Yes
*Homo sapiens*	Yes	Yes
Hydropsychidae	No	Yes
*Hydrozetes*	No	Yes
Hydrozoa	Yes	Yes
Hymenoptera	Yes	Yes
Insecta	Yes	Yes
Isotomidae	Yes	Yes
Ixodidae	Yes	Yes
Laelapidae	No	Yes
Lampyridae	No	Yes
Lasiocampidae	No	Yes
Lauriidae	No	Yes
Lepidoptera	Yes	Yes
Leptonetidae	Yes	Yes
Linyphiidae	Yes	Yes
*Longitarsus*	No	Yes
*Luciola*	No	Yes
Lumbricidae	Yes	No
*Lutzomyia*	No	Yes
Lycosidae	Yes	Yes
Lygaeidae	Yes	No
*Lymnaea*	No	Yes
Lymnaeidae	Yes	Yes
Lysianassidae	Yes	Yes
*Lysiphlebus*	Yes	No
*Macrocentrus*	No	Yes
Magnoliopsida	Yes	No
*Maiestas*	No	Yes
Malacostraca	Yes	Yes
Mammalia	Yes	Yes
Mecoptera	No	Yes
Mesostigmata	Yes	Yes
Micropholcommatidae	Yes	No
Miridae	Yes	No
Moinidae	Yes	Yes
Mollusca	Yes	Yes
Monogononta	Yes	Yes
Mucorales	Yes	Yes
Mucoromycota	Yes	Yes
Muridae	Yes	Yes
Mycetophilidae	Yes	Yes
*Mycomya*	Yes	Yes
Naididae	Yes	Yes
Naviculales	Yes	Yes
Nematoda	Yes	Yes
Nemertea	Yes	Yes
Nemesiidae	Yes	Yes
Neogastropoda	No	Yes
Neuroptera	Yes	Yes
Nymphalidae	Yes	Yes
*Oedemera*	No	Yes
Oomycota	Yes	Yes
Opiliones	No	Yes
*Opistophthalmus boehmi*	No	Yes
*Oppiella*	No	Yes
Oppiidae	No	Yes
*Oroperipatus*	No	Yes
*Orthetrum*	No	Yes
*Orthocladius*	No	Yes
Ostracoda	Yes	Yes
*Ototyphlonemertes*	No	Yes
*Oxyopes*	No	Yes
*Parabathynellidae*	Yes	Yes
Paracalliopiidae	No	Yes
*Paramecium*	No	Yes
*Paraphaenocladius*	No	Yes
*Paratanytarsus*	Yes	Yes
Perciformes	Yes	Yes
Peronosporales	Yes	Yes
*Philodromus*	Yes	Yes
Philopotamidae	Yes	Yes
*Philotrypesis*	No	Yes
Phyllodocida	Yes	Yes
*Phytomyza*	No	Yes
*Pinnularia*	Yes	Yes
Planorbidae	Yes	Yes
*Plantago*	Yes	Yes
Platyhelminthes	No	Yes
Ploima	Yes	Yes
Podocopida	Yes	Yes
Poduromorpha	No	Yes
Polychaeta	Yes	Yes
Polydesmida	Yes	Yes
Polygonaceae	Yes	No
Polyxenidae	No	Yes
Proctophyllodidae	Yes	No
*Pseudomallada*	No	Yes
*Pseudopoda*	No	Yes
Psyllidae	Yes	No
Pteromalidae	No	Yes
Ptiliidae	No	Yes
Pythiales	Yes	Yes
*Rattus*	No	Yes
*Rattus norvegicus*	Yes	Yes
*Resseliella*	Yes	No
*Rhabdias*	Yes	Yes
Rhabditida	Yes	Yes
*Rhizoglyphus*	No	Yes
Rhodophyta	Yes	Yes
Rotifera	Yes	Yes
*Salix*	No	Yes
Salticidae	Yes	Yes
Saprolegniaceae	Yes	Yes
Sarcoptiformes	Yes	Yes
Scarabaeidae	No	Yes
Scatopsidae	No	Yes
Schizomida	No	Yes
Sciaridae	No	Yes
Scincidae	No	Yes
Scolopendromorpha	No	Yes
Scorpiones	Yes	Yes
Selenopidae	Yes	Yes
*Selenops*	Yes	Yes
*Sergiolus*	Yes	No
Silphidae	Yes	Yes
*Simulium*	Yes	Yes
Streblidae	No	Yes
Strongylida	Yes	Yes
Synurophyceae	Yes	Yes
Tabanidae	No	Yes
Tachinidae	Yes	Yes
Tardigrada	No	Yes
*Tectocepheus*	No	Yes
Tenebrionidae	Yes	No
Tenthredinidae	Yes	Yes
*Tetragnatha*	Yes	Yes
Tetragnathidae	Yes	Yes
Theridiidae	Yes	Yes
Thomisidae	No	Yes
Tipulidae	No	Yes
Tomoceridae	No	Yes
Torrenticolidae	No	Yes
Trichoceridae	No	Yes
Trombidiformes	Yes	Yes
*Tuberolachnus*	Yes	Yes
*Tuberolachnus salignus*	Yes	No
Tubulinea	Yes	Yes
Uropygi	No	Yes
Veneroida	Yes	Yes
Xyelidae	Yes	No
Xystodesmidae	No	Yes
